# Simplified Procedure for Isolation and Culture of Neuronal Cells from Brains of Sickle Cell Mice

**DOI:** 10.3390/cells15110976

**Published:** 2026-05-26

**Authors:** Yugal Goel, Mya A. Arellano, Kendall O’Daniel, Donovan A. Argueta, Reina Lomeli, Naomi Lomeli, Dahlia A. Ordaz, Daniela A. Bota, Vidhya Kumaresan, Kalpna Gupta

**Affiliations:** 1 Division of Hematology and Oncology, Department of Medicine, University of California, Irvine, CA 92697, USA; ygoel@hs.uci.edu (Y.G.); arellano.mya19@gmail.com (M.A.A.); kendallodaniel@gmail.com (K.O.); daarguet@hs.uci.edu (D.A.A.); ralomeli@hs.uci.edu (R.L.); 2 Department of Neurology, School of Medicine, University of California, Irvine, CA 92697, USA; nrlomeli@hs.uci.edu (N.L.); dordaz@uci.edu (D.A.O.); dbota@hs.uci.edu (D.A.B.); 3 Chao Family Comprehensive Cancer Center, University of California, Irvine, CA 92697, USA; 4 Department of Pharmacology, Physiology & Biophysics, Boston University Chobanian & Avedisian School of Medicine, Boston University, Boston, MA 02118, USA; vidhya@bu.edu; 5 Division of Hematology, Oncology and Transplantation, School of Medicine, University of Minnesota, Minneapolis, MN 55455, USA; 6 Department of Pharmaceutical Science, University of California, Irvine, CA 92697, USA

**Keywords:** hippocampus, somatosensory cortex, neuron, sickle cell disease, pain

## Abstract

Primary neuronal cultures from the brain are critical for investigating disease-specific cellular and molecular mechanisms in mouse models. Current methods for obtaining primary cultures require embryonic brains that are affected by embryonic lethality and genotypic characterization in severe disease models such as sickle cell disease (SCD). Furthermore, these neuronal cultures require about 14 days in vitro (DIVs) for neurite outgrowth to mature. We adapted and optimized a relatively simplified and reproducible method using brains from postnatal day 1 mouse pups for isolating and culturing hippocampal and cortical neurons. This approach produces viable neurons that attach, extend neurites, and express key synaptic markers by 7 DIV and also minimizes glial outgrowth. We successfully applied this approach to isolating and culturing hippocampal and cortical neurons from the brains of one-day-old (P1) pups of humanized transgenic homozygous BERK sickle cell and control mice. Morphological observations at 3, 7, and 14 DIVs demonstrated robust neuronal attachment, neurite outgrowth, and overall structural development in both male and female hippocampal and cortical neurons. Neurons in culture expressed key markers including neuronal nuclear protein (NeuN/Rbfox3), neurofilament 200 (NF200), microtubule-associated protein 2 (MAP2), vesicular glutamate transporter 1 (VGLUT1), postsynaptic density protein 95 (PSD 95), and glutamate N-methyl-D-aspartate receptor subunit 2B (GluN2B). Notably, male SCD hippocampal neurons evinced a higher density of PSD 95 puncta on dendritic spines compared to controls on 7 as well as 14 DIVs. Incubation of male hippocampal neurons in a sickle cell-like microenvironment with TNF-α and heme further increased the density of PSD 95 puncta and colocalization of GluN2B with PSD 95, supporting the utility of this culture system for examining disease-relevant structural and molecular responses. This optimized culture system provides a simplified and reproducible platform to investigate the mechanisms involving neuronal dysfunction in challenging mouse models of brain disorders.

## 1. Introduction

The genetically inherited condition, sickle cell disease (SCD), is accompanied by cognitive impairment and chronic pain [1,2,3]. Humanized homozygous transgenic mice with SCD show both cognitive deficits and chronic hyperalgesia [4,5,6,7,8,9]. The hippocampal and cortical regions of their brains show increased oxidative stress, demonstrated by increased reactive oxygen species (ROS), lipid peroxidation, and protein carbonylation [10]; in addition, neuropathologic changes are observed in their hippocampus and cerebellum [7]. Thus, a critical unmet need is to understand the mechanisms underlying hippocampal pathology that contribute to pain and cognitive dysfunction in SCD.

The hippocampus is linked to cognitive impairment associated with pain, and mechanistic inconsistencies underlying its impaired function have been observed across different studies on various processes, such as dendritic complexity [11,12,13]. Some studies report dendritic atrophy and reduced spine density in the hippocampus during chronic pain [14,15], whereas others describe compensatory increases in dendritic branching or region-specific differences across CA1, CA3, and dentate gyrus [16]. Similarly, reports of hippocampal long-term potentiation (LTP) show conflicting outcomes, with studies demonstrating either impaired synaptic plasticity or paradoxical increases in excitatory signaling depending on the pain model and duration [15,16]. Variability has also been noted in neuroinflammatory profiles, oxidative stress levels, and excitatory–inhibitory balance, contributing to divergent interpretations of how pain alters hippocampal structure and function. This could be due to differences arising from the pathobiology of the disease and/or environmental or other stressors. Structural and functional differences in the hippocampus have been observed in male and female rats with chronic neuropathic pain [17].

In addition to the hippocampus, the cerebral cortex plays a vital role in cognitive and sensory processing. Among its subdivisions, the somatosensory cortex is essential for interpreting touch, pressure, temperature, and pain (nociception) [18]. It is a key site for the central integration of peripheral pain signals, and changes in this region are well-documented in chronic pain states, including neuropathic and inflammatory pain, and are thought to contribute to central sensitization and maladaptive pain processing [19,20]. Central sensitization is known to exist in persons with SCD based on neuroimaging and quantitative sensory testing [21,22] as well as in transgenic Berkeley (BERK) sickle cell mice expressing human sickle cell hemoglobin (HBSS) [23]. Considering the significance of central sensitization in sickle cell pain, it is vital to understand the neuronal mechanisms contributing to it.

To address pain and cognitive dysfunction, we sought to develop an in vitro system to examine their mechanisms in SCD. A major challenge with existing protocols to isolate and culture primary neurons was the use of embryonic tissue. The reason for this is that female homozygous sickle cell mice do not breed well. Homozygous (HbSS) males are bred with hemizygous (HbAS) females, which produce about 2/3 hemizygous and about 1/3 homozygous pups in a litter. Fetal absorption and in utero embryonic mortality are common, and the offspring are extremely fragile with high mortality. Blood from the pups must be phenotyped to select the homozygous sickle cell offspring, which is not possible in embryos. Therefore, we developed a protocol that allows the use of phenotype-confirmed postnatal (P1) pups instead of embryos. We adapted and optimized a relatively simplified and reproducible method to isolate and culture hippocampal and cortical neurons from postnatal, one-day-old fragile HbSS sickle cell and control HbAA mice. We show that our improved and simplified procedure successfully yields sustainable hippocampal and cortical neurons on postnatal day 1 from both sexes of transgenic HbSS-BERK ‘sickle’ cell mice expressing >99% human HbS. This approach produces neurons that attach, extend neurites, and express synaptic markers by 7 DIVs, showing maturation comparable to or faster than timelines reported for embryonic or early postnatal culture protocols [24,25]. Both hippocampal and cortical neurons are maintained in cultures for 14 days and show typical neuronal morphology with dendritic spines and express characteristic neuronal markers, such as NeuN, neurofilament 200 (NF200), microtubule-associated protein 2 (MAP2), vesicular glutamate transporter 1 (VGLUT1), postsynaptic density 95 (PSD 95) and N-methyl D-aspartate receptor subtype 2B (GluN2B). We provide evidence that these cells can be tested in culture using mechanism-based therapeutic agents under a sickle cell microenvironment replete with free heme and inflammation using tumor necrosis factor alpha (TNF-α), thus providing a critical tool to examine the mechanisms underlying cognitive dysfunction and pain. This method can also be applied to mouse models of different diseases.

## 2. Materials and Methods

### 2.1. Mice

To establish primary hippocampal and cortical neuronal cultures, we utilized male and female postnatal day 1 pups from humanized, homozygous transgenic HbSS-BERK ‘sickle’ cell mice expressing >99% HbS, which is a characteristic feature of SCD. For the control group, we used HbAA-BERK mice pups expressing normal human hemoglobin (HbA), without murine α- or β-globins on the same mixed genetic background of HbSS-BERK mice. All pups were phenotyped by cellulose acetate electrophoresis of the blood, and those homozygous for sickle cell Hb (HbSS) were used, and those expressing only human HbA without sickle cell Hb (HbAA) were used. All mice and pups were housed in an AAALAC-approved facility, in a 12 h dark/light cycle, and provided with food and water ad libitum. All animal experiments were performed after prior approval from the IACUC (protocol #1618945) in accordance with guidelines from the National Institutes of Health (NIH).

### 2.2. Reagents Used in Neuronal Isolation and Culture

The following reagents were used for neuronal isolation and maintenance in culture: Neurobasal™ Medium (Gibco, Thermo Fisher Scientific, Waltham, MA, USA, #21103049), B27 Supplement (Gibco, #17504044), L-Glutamine (200 mM; Gibco, #25030081), Glutamax (L-alanyl-L-Glutamine dipeptide; Gibco, #35050061), Penicillin–Streptomycin (10,000 U/mL; Gibco, #15140122), Fetal Bovine Serum (FBS; Gibco, #A3840102), substrate coating reagents such as poly-D-lysine hydrobromide (Sigma-Aldrich, St. Louis, MO, USA, #P7280) and laminin (Corning, Corning, NY, USA, #354232). Enzymatic dissociation was performed using papain (Thermo, #416760100) combined with the papain/Trypsin inhibitor (Sigma, #T-9253). Pharmacological agents used during culture and synaptogenesis modulation included DL-2-Amino-5-phosphonopentanoic acid (APV; Sigma, #A-5282) and Cytosine β-D-arabinofuranoside hydrochloride (Ara-C; Sigma, #C6645). Dissection and plating solutions were prepared with Bovine Serum Albumin (BSA; Sigma, #A9418), L-Cysteine (Sigma, #C7352), Ethylenediaminetetraacetic acid (EDTA; Sigma, #E6758), Sodium chloride (NaCl; Sigma, #S5886), Potassium chloride (KCl; Sigma, #P5405), Sodium dihydrogen phosphate (Na_2_HPO_4_; Sigma, #S5136), Potassium dihydrogen phosphate (KH_2_PO_4_; Sigma, #RDD037), HEPES (Sigma, #H0887), and D-glucose (Sigma, #G7021). Cultureware included clear tissue culture-treated 6-well and 24-well plates (Corning, #3516 and #3526) and round glass coverslips, 15 mm and 12 mm in diameter (Chemglass Life Sciences, Vineland, NJ, USA, #CLS1760015 and #CLS1760012).

### 2.3. Step-by-Step Protocol for Primary Culture of Hippocampal and Cortical Neurons from Newborn Mice (P1)

**Scheme 1:**
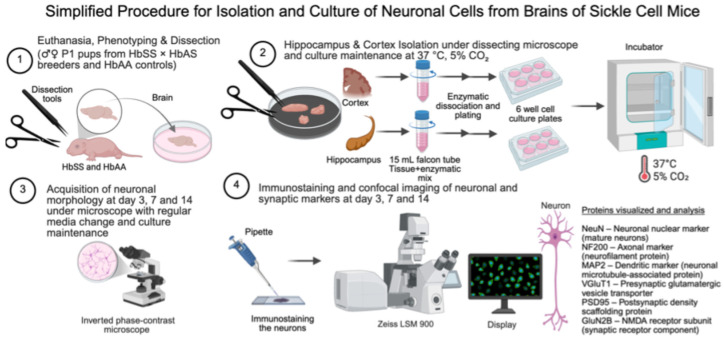
Schematic overview of the isolation, culture, and analysis of hippocampal and cortical neurons from postnatal day 1 control (HbAA) and sickle cell (HbSS) mice. The workflow includes brain dissection, enzymatic dissociation, neuronal plating and maintenance, and downstream morphological and immunocytochemical analyses.

The overall workflow for neuronal isolation, culture, and downstream analyses is summarized in Scheme 1.

#### Reagent Preparation for Dissection and Primary Neuron Culture

- Laminin: A 200 µg/mL stock solution was prepared by dissolving laminin (1 mg) in 1× HBSS (5 mL). Aliquots of 100 μL were made and frozen at −80 °C for up to 6 months.
- Poly-D-lysine: A 2 mg/mL stock solution was prepared by dissolving poly-D-lysine (5 mg) in ddH_2_O (2.5 mL). Aliquots of 110 µL were made and frozen at −20 °C. A 20 µg/mL working solution was prepared by diluting 2 mg/mL poly-D-lysine (100 µL) in 1× HBSS (10 mL). Approximately 1.2 mL was aliquoted per 12 coverslips and kept on ice until use. The remainder was stored at 4 °C for up to one week.
- Dissection Solution (DS): NaCl (4.003 g), KCl (0.201 g), Na_2_PO_4_ (0.012 g), KH_2_PO_4_ (0.015 g), HEPES (1.180 g), D-glucose (3.000 g), and sucrose (7.496 g) were combined in diH_2_O. The pH was adjusted to 7.40 with NaOH, and the final volume was brought to 500 mL with diH_2_O. The solution was sterile-filtered using a 0.2 µm vacuum filter and stored at 4 °C.
- The Neurobasal Medium + B27 (NBM/B27): Neurobasal™ Medium (250 mL) and B27 Supplement (5 mL) were combined, sterile-filtered through a 0.2 µm filter, and stored at 4 °C for up to 2 months.
- Conditioned Plus Media (CM+): B27 Supplement (1 mL), GlutaMAX (125 µL), L-Glutamine (125 µL), Penicillin–Streptomycin (500 µL), and FBS (5 mL) were combined and brought to a final volume of 50 mL with Neurobasal™ Medium. The solution was sterile-filtered through a 0.2 µm filter and stored at 4 °C for up to 2 weeks.
- Conditioned Minus Media (CM−): B27 Supplement (1 mL), GlutaMAX (125 µL), and Penicillin–Streptomycin (500 µL) were combined and brought to a final volume of 50 mL with Neurobasal™ Medium. The solution was sterile-filtered through a 0.2 µm filter and stored at 4 °C for up to 2 weeks.
- APV: A 5 mM APV stock was prepared by dissolving APV (9.86 mg) in ddH_2_O (10 mL). The solution was sterile-filtered through a 0.2 µm filter. Aliquots of 120 μL and 500 µL were stored at −20 °C.
- Ara-C: A 1 mM Ara-C stock was prepared by dissolving Ara-C (27.97 mg) in ddH_2_O (100 mL). The solution was sterile-filtered through a 0.2 µm filter. Aliquots were prepared and stored at 4 °C or −20 °C for up to 6 months.
- Papain: A 1 U/µL stock was prepared by dissolving papain (5 mg) in diH_2_O (5 mL). The solution was sterile-filtered through a 0.2 µm filter and kept on ice. It may be stored at 4 °C for up to one week.
- Enzyme Stock Preparation: In total, 5 mM APV (500 µL), L-cysteine (6 mg), and EDTA (9.3 mg) were combined and brought to a final volume of 50 mL with the dissection solution. The mixture was vortexed thoroughly until dissolved, then sterile-filtered through a 0.2 µm filter. Aliquots of 4 mL for hippocampal neurons and 12 mL for cortical neurons were stored at −20 °C.
- Complete Enzyme Solution (ES): Immediately before use, one aliquot of enzyme stock solution was thawed and supplemented with 50 units of papain to prepare the complete enzyme solution. The solution was then activated in a 37 °C water bath for 30 min prior to tissue digestion.
- Bovine Serum Albumin/Papain Inhibitor Stock (BSA/TI): In total, 10 mL of dissection solution was added to a large beaker, and BSA (1 g) was sprinkled evenly on top. The solution was allowed to dissolve completely without agitation or vortexing. Once dissolved, the Trypsin Inhibitor (1 g) was added in the same manner and allowed to dissolve fully. The final solution was sterile-filtered through a 0.2 µm filter, and aliquots of 400 µL were stored at −20 °C.
- High Inhibitor (HI) Solution: The HI solution was freshly prepared by combining dissection solution (DS, 3.0 mL), BSA/TI stock (300 µL; final concentration 10 mg/mL), and 5 mM of APV stock (30 µL; final concentration 50 µM). The mixture was gently mixed, and 1.5 mL was aliquoted into two 15 mL conical tubes and kept on ice until use.
- Low Inhibitor (LI) Solution: The LI solution was freshly prepared by combining DS (8.0 mL), BSA/TI stock (80 µL; final concentration 1 mg/mL), and 5 mM APV stock (80 µL; final concentration 50 µM). The mixture was gently mixed, and 2.5 mL was aliquoted into three 15 mL conical tubes and kept on ice until use.

### 2.4. Neonatal Pup Phenotyping

- Sex: The sex of neonatal pups was determined on postnatal day 1 by visual inspection of the anogenital region. Males were identified based on a greater anogenital distance and the presence of a distinct pigmentation spot above the urethral opening, indicative of developing scrotal skin. This non-invasive method is reliable for early sex differentiation in mouse pups.
- Homozygous sickle cell pups: The sickle cell pups (HbSS-BERK mice) were bred and phenotyped using cellulose acetate electrophoresis, as described, to distinguish between homozygous and hemizygous pups. Only pups homozygous for HbS were used [26,27].

### 2.5. Surface Preparation of Coverslips for Neuronal Seeding

- Sterilization: Round glass coverslips (15 mm or 12 mm) were sterilized by autoclaving. Under sterile conditions in a biosafety cabinet, each coverslip was dipped in 70% ethanol and set upright in a sterile Petri plate to dry. Once dried, coverslips were centered flat in the 6/24 cell culture plate wells to ensure an even coating and prevent solution runoff. Care was taken to avoid contact between coverslips and the walls of the well.
- Coating Solution: Prior to use, a laminin aliquot was thawed at 4 °C and kept on ice. A working solution of poly-D-lysine (20 µg/mL) was prepared by diluting the 2 mg/mL stock (100 µL) into 10 mL of 1× HBSS. To prepare the final coating solution, 100 µL of laminin stock was diluted into a 1.1 mL poly-D-lysine solution.

Within 10 min of mixing, 100 µL of this coating solution was pipetted gently onto the center of each coverslip. The coated plates were stored at room temperature for at least 2 h or overnight prior to cell plating.

### 2.6. Dissection of Neonatal Brain for Hippocampal and Cortical Collection

- Inside the biosafety cabinet, one 60 mm Petri dish containing 3 mL of ice-cold dissection solution was prepared for brain sample collection, along with two sterile tubes containing 3 mL of ice-cold dissection solution for hippocampus sample collection and 9 mL for cortical region sample collection. Two enzyme solution (ES) aliquots were thawed, and 50 units of papain were added to each to prepare the complete ES, which was then incubated in a 37 °C water bath for 30 min to ensure optimal papain activity.
- Pups were euthanized by placing them inside a CO_2_ chamber with a fill rate of 70% displacement of the chamber volume per minute. The flow rate was adjusted based on chamber size, and pups were monitored until respiration ceased.

Note: Dissection was carried out in a disinfected area within the flame-sterile zone near the Bunsen burner to maintain aseptic conditions.

- Once the pups were unconscious, their bodies were disinfected with 70% ethanol using a spray bottle. Euthanasia was confirmed, followed by decapitation using large straight scissors.

Note: Trunk blood was collected immediately after decapitation of pups into EDTA-coated tubes for sickle cell phenotyping.

- A midline cranial incision was made through the skin with small, straight scissors under aseptic conditions. Another midline incision was made through the skull, followed by two small incisions, one near each eye. Using forceps, the skull was carefully peeled back to expose the brain. The brain was removed from the skull by gently detaching the occipital lobe and underlying nerves with small forceps. Once extracted, the brain was placed in a Petri dish containing ice-chilled dissection solution.
- Using a dissecting microscope, the hippocampus was isolated from the rest of the brain by making a midline incision between the two hemispheres with fine forceps and small straight scissors. While the cerebellum was held with forceps, another pair of forceps was used to carefully ‘unroll’ the cortex from the hemisphere until the hippocampus became visible. The hippocampus was then removed using small straight scissors, and the procedure was repeated for the second hemisphere.
- Meninges were carefully removed and discarded. Following hippocampal removal, the frontal, parietal, and occipital cortices were carefully separated from the underlying white matter using fine forceps under a dissecting microscope.
- Each hippocampus (from both hemispheres) was cut into 3–4 pieces in a 60 mm Petri dish containing ice-cold dissection solution using fine scissors. The hippocampal tissue was transferred into a pre-labeled tube containing 3 mL of dissection solution. Separately, the dissected cortical regions (cut into pieces) were transferred into another tube containing 9 mL of dissection solution. Both tubes were kept on ice before proceeding to the culture hood for further processing.

### 2.7. Cell Dissociation and Plating

- After the 30 min incubation of complete ES at 37 °C, the enzyme solution with papain was filtered through a 0.2 μm filter into two sterile tubes.
- The tissues were transferred into the complete ES in designated sterile tubes for the hippocampus and cortex using a transfer pipette, allowing them to gently slide down via gravity while minimizing the carryover of the dissection solution.
- Under sterile conditions, tubes containing hippocampal and cortical tissues immersed in complete ES were incubated at 37 °C for 25 min.
- HI and LI solutions were prepared in 15 mL conical tubes and kept on ice during incubation.
- Tissues were transferred with a sterile transfer pipette before a sequence of washes was performed in individual tubes by gently swirling up and down ~3 times, using as little residual solution as possible. After every wash, the tube was placed back on ice and the tissue was allowed to settle to the bottom of the tube for ~1 min before proceeding to the next wash as follows in the sequence described below.
  ✓ One wash with dissection solution (4 mL hippocampal; 12 mL cortical);
  ✓ Two washes with HI Solution (2 mL hippocampal; 6 mL cortical)
  ✓ Two washes with LI Solution (3 mL hippocampal; 9 mL cortical);
  ✓ One wash with NBM/B27 Media (4 mL hippocampal; 12 mL cortical);
  ✓ One wash NBM/B27 Media (1.5 mL hippocampal; 4.5 mL cortical).
- The cell suspension was visually triturated using a manual micropipette with a decreasing tip diameter:
  ✓ This was performed ~15–20 times with a 5 mL sterile serological pipette.
  Note: The pipetting should be gentle, and a consistent force should be used to initiate dissociation without shearing cells. Frothing or bubbling of the solution was avoided.
  ✓ This was performed ~20 times with a 1 mL pipette tip.
  Note: The tip was slightly cut to widen the bore, reducing mechanical stress and helping prevent cell lysis.
  ✓ This was performed ~15 times with a 20–200 μL pipette tip.
  Note: This step helped break up finer clumps. Pipetting was performed slowly and steadily to avoid the formation of bubbles or creation of shear stress.

- The suspension was left undisturbed for ~3 min to allow larger pieces of tissue to settle to the bottom.
- Immediately before plating, the coating solution was aspirated, and coverslips were gently rinsed three times with 1× HBSS (100 µL per wash) using just enough volume to cover only the coated central area of each coverslip, for 1 min per wash. After the final rinse, the HBSS was completely aspirated, and 100 µL of cell suspension was loaded onto each coverslip at 1 × 10^4^ live cells/coverslip.

Note: Prior to plating, cell viability was assessed using Trypan Blue exclusion and a hemocytometer. Only live (unstained) cells were counted to accurately adjust the cell suspension concentration.

- Coverslips were incubated at 37 °C and 5% CO_2_ for at least 2–3 h to allow cells to adhere.
- Warm CM+ was added to each well (500 µL for 24-well plates and 2 mL for 6-well plates).
- At day 2, cultures were treated for 24 h with Ara-C to inhibit glial growth by diluting Ara-C stock in CM− to achieve a final concentration of 2 µM.
- After incubation, 80% of the media was removed and replaced with fresh CM− (250 µL for 24-well plates and 1 mL for 6-well plates).
- The medium was refreshed twice weekly by replacing half of the volume with fresh, warmed CM−.

### 2.8. Treatments

TNF-α, 1 ng/mL (#AAJ67162EXE, Thermo Fisher Scientific, Waltham, MA, USA) was prepared in sterile saline and diluted to its final concentration in culture medium. Fresh hemin (2.67 mM) was prepared by mixing 10 mg of hemin chloride (Frontier Specialty Chem, Logan, UT, USA, #H651-9), 10 mg of D-sorbitol, and 6.9 mg of sodium carbonate in 5.7 mL of sterile saline for 60 min in the dark [28]. Hemin was diluted to a final concentration of 40 μM in the culture medium for incubating cells after filtering with a 0.22 μm filter. Similar to previously described sickle cell microenvironment stimulation protocols [28], cells were treated with freshly prepared TNF-α for 2 h, followed by co-treatment with TNF-α and hemin for an additional 2 h. Cells were thoroughly washed with sterile phosphate-buffered saline (PBS), and then fixed in 2% paraformaldehyde (Thermo Fisher, #AC416780250) in PBS for 10 min at room temperature (RT).

### 2.9. Laser Scanning Confocal Microscopy (LSCM) for Neuron-Specific Markers

Primary hippocampal and cortical neurons from postnatal day 1 HbAA and HbSS (male or female) mice were maintained at 37 °C. Cells were fixed as described above at 3, 7, and 14 days in vitro (DIVs) with 2% paraformaldehyde in phosphate-buffered saline(Sigma-Aldrich, #158127) for 10 min at room RT and permeabilized with ice-cold 0.1% Triton X-100 (Sigma-Aldrich, #T8787) for 2 min; they were then blocked for 30 min with 3% donkey serum in PBS and incubated overnight at 4 °C with combinations of primary antibodies (Supplemental Table S1): goat anti-RBFOX3/NeuN (1:200, Novus Biological, Centennial, CO, USA, #NBP3-05554), chicken anti-NF200 (1:3000, Neuromics, Edina, MN, USA, #CH22104), guinea pig anti-postsynaptic density (PSD) 95 (1:500, Alomone Labs, Jerusalem, Israel, #APZ-009-GP), rabbit anti-GluN2B (1:500, Alomone Labs, #AGC-003), chicken anti-MAP2 (1:2000, Thermo Fisher, #PA1-1005), and rabbit anti-VGLUT1 (1:300, Cell Signaling Technology, Danvers, MA, USA, #47181). This was followed by 1 h of incubation at RT with secondary antibodies: Cy™2 AffiniPure donkey anti-goat (1:200, Jackson ImmunoResearch, West Grove, PA, USA, #705-225-147), Cy™5 AffiniPure donkey anti-chicken (1:200, Jackson ImmunoResearch, #703-175-155), Cy™5 AffiniPure donkey anti-guinea pig (1:200, Jackson ImmunoResearch, #706-175-148), and Cy™2 AffiniPure donkey anti-rabbit (1:200, Jackson ImmunoResearch, #711-225-152). Coverslips containing the cells were mounted onto glass microscope slides with ProLong™ diamond antifade mounting media (Thermo Fisher Scientific, #P36962) and nuclear counterstain 4′, 6-diamidino-2-phenylindole (DAPI). Appropriate negative controls for immunostaining were also used for each set of staining. Images were acquired on a LSCM (Zeiss LSM 900, Carl Zeiss AG, Oberkochen, Germany), using a plan-apochromat 20×/NA: 0.8 M27 objective lens, 9-tile scans of 0.222 µm × 0.222 µm fields of view (arranged 3 × 3), and Z-stacks of 10 × 0.5 µm images. ImageJ software was used to process the images (version 1.54r; National Institutes of Health, Bethesda, MD, USA). Representative images of cells isolated from 3 to 5 mice of each genotype are presented.

### 2.10. Evaluation of PSD 95 and GluN2B

Dendritic PSD 95 puncta formation was evaluated as previously described with modifications to optimize primary hippocampal cultures from HbSS and HbAA mice [29,30]. Cells immunolabeled for PSD 95 were evaluated for dendritic spine formation by quantifying the number of PSD 95 puncta in 20 µm segments of dendrites extending from the soma. Individual puncta were considered as separate spines and not adjusted for puncta size. Dendrites of the same order and puncta counts from the same distance relative to the soma were directly compared. It was required to combine 9 tile scans of 0.222 µm × 0.222 µm fields of view (arranged 3 × 3) with Z-stacks of 10 × 0.5 µm images on a laser scanning confocal microscope (Zeiss LSM 900, Carl Zeiss AG) using a plan-apochromat ×63 magnification objective lens and tile feature to acquire the images of individual soma and dendrites.

PSD 95 and GluN2B colocalization were quantified using the Coloc2 plugin in FIJI/ImageJ, as described by Dunn et al. (2011) [31]. Pearson’s correlation coefficient (PCC) was used to assess the degree of spatial overlap between the two fluorescence channels. PCC values ranged from −1 to +1, where +1 indicates perfect correlation (complete overlap), 0 indicates random distribution, and −1 indicates complete exclusion. In synaptic imaging, higher PCC values reflect enhanced spatial proximity of postsynaptic scaffolding proteins and NMDA receptor subunits, suggesting increased synaptic maturity. Dendritic regions of interest (ROIs) were manually selected, focusing on secondary or tertiary dendrites located 20–40 µm from the soma. Background subtraction was applied, and automatic Costes thresholding was used to ensure consistent and objective measurements. At least 9 ROIs representing each image from 3 mice (3 acquisitions per mouse) were obtained per condition. For statistical analyses, ‘*n*’ was 3, not 9, because 3 values from each mouse were averaged, and the mean was used to obtain one value per mouse.

### 2.11. Statistics

Data are shown as the mean ± SEM and analyzed with two-way ANOVA and Tukey’s multiple comparisons post hoc tests (GraphPad Prism software, version 10.1.324; GraphPad Software, Boston, MA, USA). *p*-values < 0.05 are considered statistically significant.

## 3. Results

### 3.1. Developmental Progression of Cortical and Hippocampal Neurons in Control and Sickle Cell Models Across 3, 7, and 14 DIVs

Neuronal cultures were established from both male and female pups. Morphological and neuronal marker analysis was performed on cells from both sexes. Analysis of synaptic proteins PSD 95 and GluN2B was only performed under a sickle cell microenvironment for neurons derived from male pups as a proof of concept that these neuronal cells are responsive to the microenvironment.

Primary cortical and hippocampal neurons were isolated from HbAA and HbSS neonatal mice (P1) of both sexes to evaluate developmental changes across genotypes and brain regions. Live cultures were observed and analyzed at DIVs 3, 7, and 14 to qualitatively assess morphological maturation, including soma density, neurite outgrowth patterns and network complexity.

**Figure 1:**
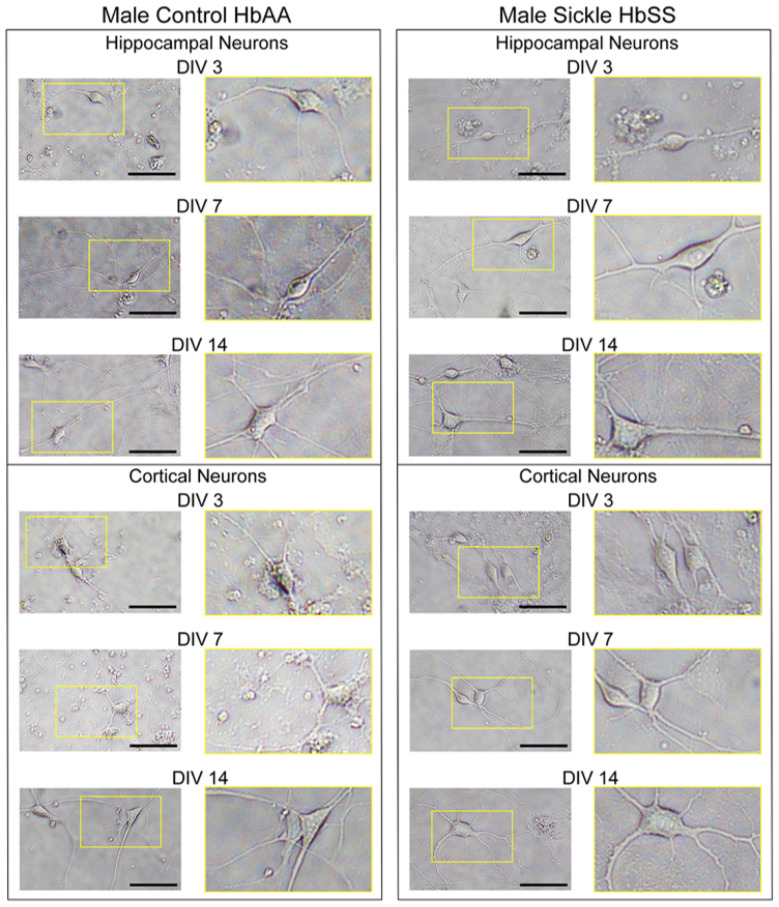
Morphological maturation of hippocampal and cortical neurons from control and sickle cell male mice across DIVs 3, 7, and 14. Representative brightfield images of live primary hippocampal and cortical neurons isolated from control and sickle cell male neonatal mice (postnatal day 1) are shown. Compared with DIV3, neurons at DIV14 qualitatively showed more extensive axonal processes across both genotypes in both hippocampal and cortical neurons. Magnification ×20; scale bar: 50 µm. Each image represents cells from 3 different mice per genotype. Additional representative images from male and female cultures are provided in Figure S1. Abbreviations: DIV, days in vitro; HbAA, homozygous mice expressing normal human hemoglobin A; HbSS, homozygous sickle cell mice expressing human sickle cell hemoglobin.

**Figure 2:**
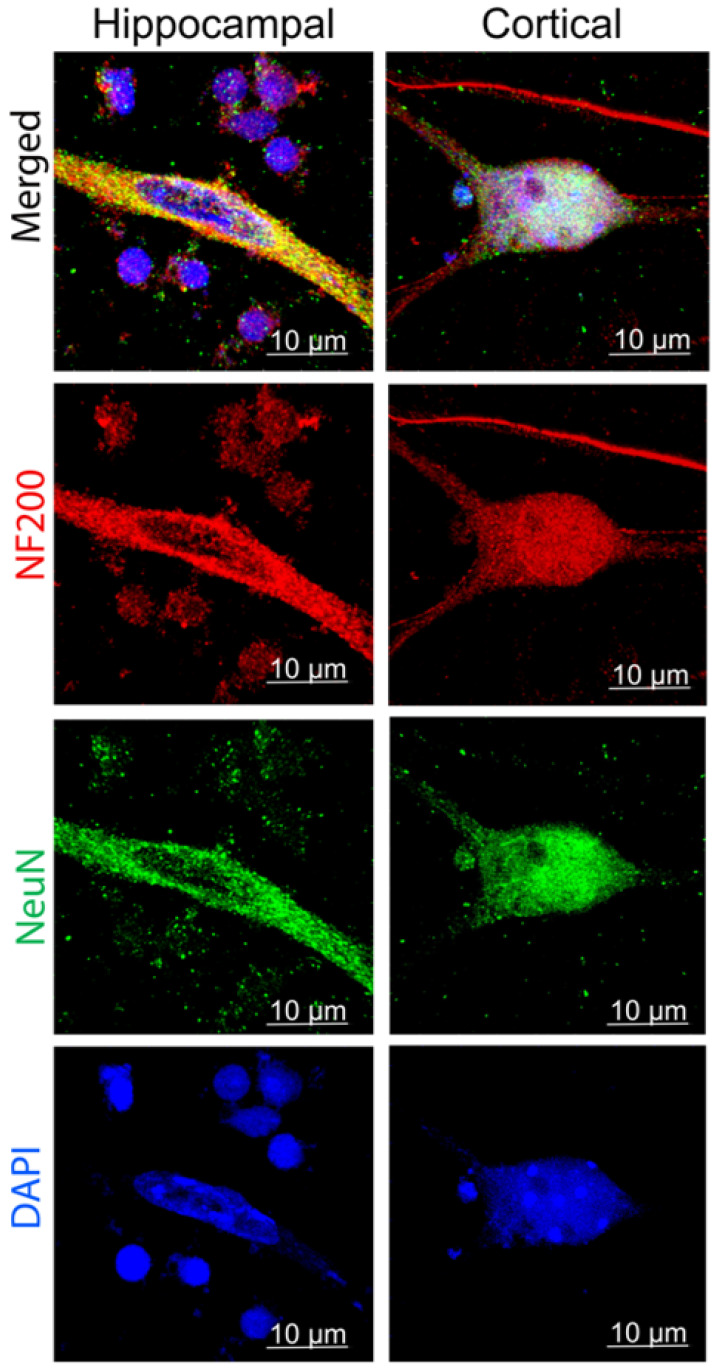
NeuN and NF200 immunoreactivity of neuronal cells at DIV 7. Primary hippocampal and cortical neurons were isolated from male sickle cell neonatal mice (postnatal day 1) and immunostained for NeuN (green), which is a neuronal marker, and NF200 (red), a neurofilament protein marking axons, and counterstained with DAPI (blue) to visualize nuclei. Each image represents reproducible images from three different mice, with 3 images acquired per mouse (9 images/condition). The complete image set for control and sickle cell male and female hippocampal and cortical neurons at DIVs 3, 7, and 14 is provided in Figure S2. Confocal images were acquired using ×63 objective with ×2.3 digital zoom and z-stacks (0.5 µm step size). Scale bar: 20 µm. Abbreviations: DIVs, days in vitro; NeuN, neuronal nuclei (RBFOX3); NF200, neurofilament 200 kDa.

- DIV 3—Initial Attachment and Onset of Neurite Outgrowth (Figure 1): By DIV 3, cultured neurons showed successful attachment and early neurite outgrowth across all groups, including male and female, control and sickle-cell-derived neurons. While some variation in cell spreading and morphology of neurites was noted between cortical and hippocampal neurons, the cultures consistently displayed features of neuronal structure. These results suggest that the protocol is broadly applicable across sex and genotypes, although further studies will be needed to systematically characterize morphological differences. These morphological trends in hippocampal and cortical neurons were further supported by the presence of neuronal markers (Figure 2). Control and sickle cell neurons from male hippocampal and cortical cultures showed robust NeuN+ staining and emerging NF200+ neurites, indicative of healthy neuronal cells.
- DIV 7—Neurite Expansion and Early Network Formation (Figure 1): By DIV 7, neuronal maturation advanced substantially in control and sickle-cell-derived cultures. Qualitatively, cortical neurons demonstrated extensive branching with the emergence of neurite interconnections, while hippocampal neurons extended processes. Sickle cell neurons began to show more apparent variability in soma size and neurite density, especially in hippocampal cultures. Further, control and sickle cell neurons from male hippocampal and cortical cultures showed robust NeuN+ expression and NF200+ expression in neurites, indicating the preservation and specificity of neuronal integrity (Figure 2).
- DIV 14—Network Maturation and Structural Differentiation (Figure 1): At DIV 14, neuronal networks in control cultures showed dense and highly branched neurite webs observed in both cortical and hippocampal regions. Cortical neurons showed rich arborization and complex interconnectivity, while hippocampal neurons displayed organized, long projections that formed bundled tracts. Control and sickle cell hippocampal and cortical neurons maintained constant NeuN+ expression and NF200+ neurite networks at DIV 14 (Figure 2).

### 3.2. MAP2 and VGLUT1 in Sickle Cell vs. Control Hippocampal Neurons Across Developmental Timepoints

**Figure 3:**
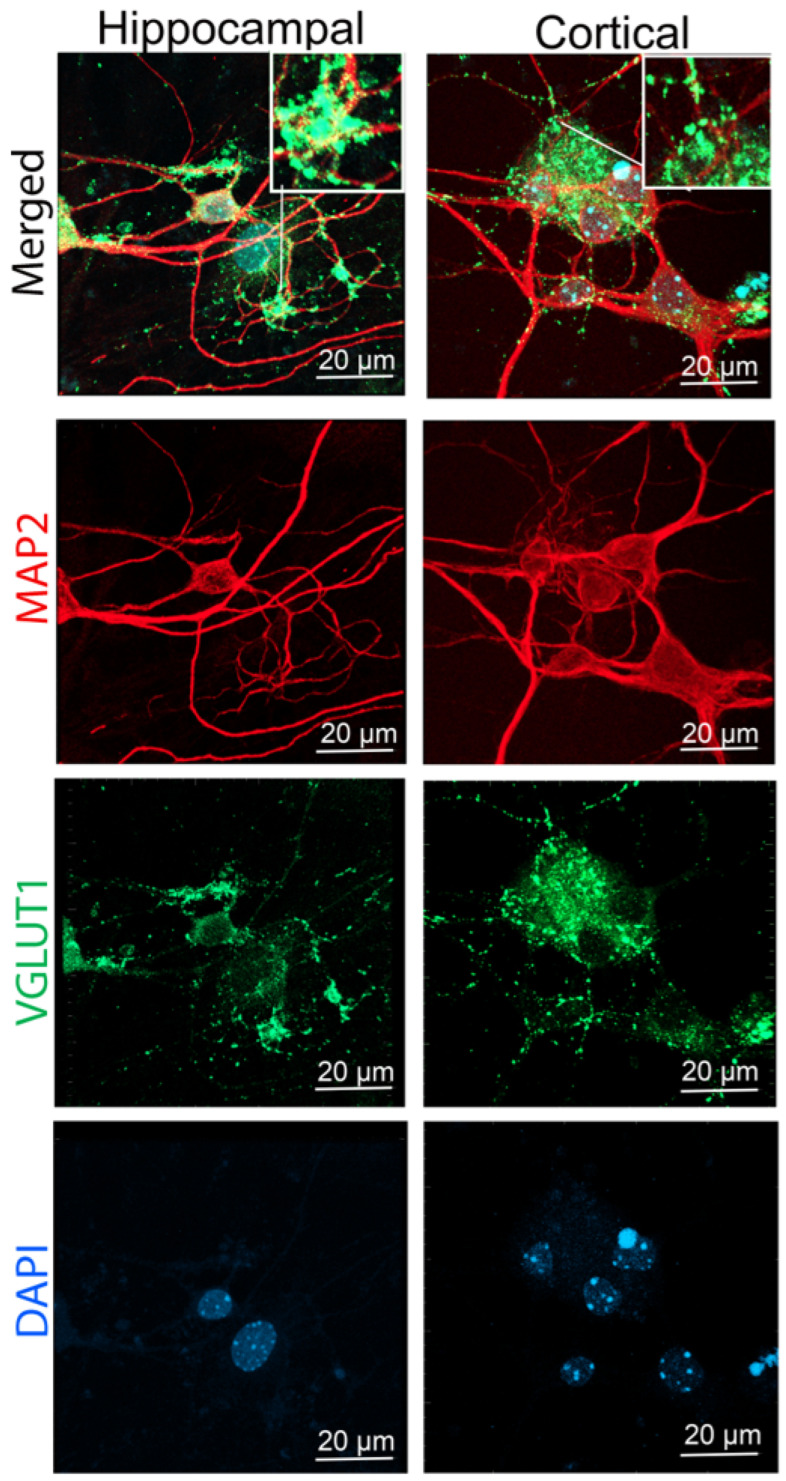
MAP2 and VGLUT1 immunoreactivity of hippocampal and cortical neurons at DIV 7 from male sickle cell mice. Primary hippocampal and cortical neurons were isolated from male sickle cell neonatal mice (postnatal day 1). Neurons were immunostained for VGluT1 (green), a presynaptic marker of glutamatergic vesicles, and MAP2 (red), a dendritic marker, at DIV 7. Nuclei were counterstained with DAPI (blue). Insets show higher magnification views highlighting VGLUT1 puncta along MAP2-positive neuronal processes. Confocal imaging was performed using a ×63 oil immersion objective with a 0.5 µm z-step. Each image represents reproducible images from three different mice (*n* = 3 mice), and 3 images acquired per mouse (9 images/condition). The full image set, including control and sickle cell neurons at DIV 3, 7, and 14, is provided in Figure S3. Abbreviations: DIVs, days in vitro; MAP2, microtubule-associated protein 2; VGLUT1, vesicular glutamate transporter 1.

To assess neuronal development and excitatory synapse formation, hippocampal and cortical neurons isolated from male control and sickle cell mice were immunostained for MAP2 (red), VGLUT1 (green), and DAPI (blue) at DIV 3, DIV 7, and DIV 14 (Figure 3). MAP2 staining was present at all timepoints, confirming dendritic markers and demonstrating progressive elaboration of neuronal processes across the culture period. VGLUT1 immunoreactivity was detected from DIV3 onward in association with neuronal processes, which is consistent with developing excitatory synaptic organization. The persistence of MAP2 and VGLUT1 staining from DIV 3 through DIV 14 provides a means to evaluate continuous neuronal viability, dendritic maturation, and establishment of excitatory synaptic contacts during development in vitro. Merged images further show the close spatial relationship between dendritic structure and presynaptic markers, which suggests ongoing synaptic organization over time.

### 3.3. Genotype, Development, and Sickle Cell Microenvironment Modulate PSD 95 and GluN2B Colocalization in Cultured Hippocampal Neurons

**Figure 4:**
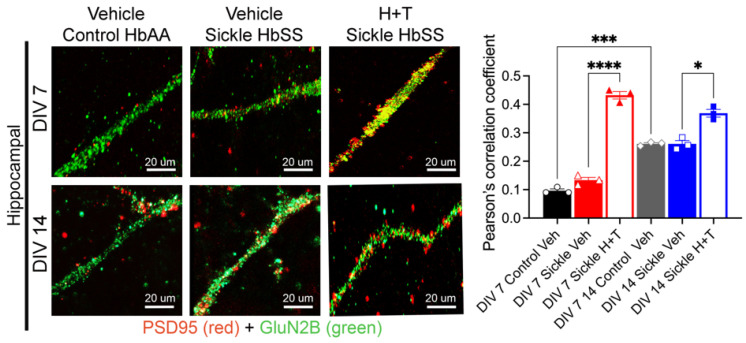
GluN2B and PSD 95 immunoreactivity in dendritic spines of control and sickle cell mice-derived hippocampal neurons at DIV 7 and DIV 14. Primary hippocampal neurons were isolated from male control and sickle cell neonatal mice (postnatal day 1) and cultured under the following conditions: vehicle-treated control, and vehicle- or hemin + TNF-α (H+T)-treated sickle cell mice. Neurons were immunostained for PSD 95 (red) and GluN2B (green) at DIV 7 and DIV 14. Confocal microscopy using ×63 oil immersion objective (z = 0.5 µm). The yellow signal indicates the colocalization of GluN2B and PSD 95 puncta, representing potential postsynaptic assembly of NMDA receptor subunits at excitatory synapses. Each image represents reproducible images from three different mice (*n* = 3 mice/genotype), with 3 images acquired per mouse (9 images/condition). The graph represents quantification of colocalization of PSD 95 and GluN 2B using Pearson’s correlation coefficient (ImageJ). A quantification mean of 3 different images per mouse was used as a single value and values from 3 mice per condition were used for analysis. Data are presented as mean ± SEM and analyzed using two-way ANOVA with Tukey’s multiple-comparisons test (* *p* < 0.05, *** *p* < 0.001, **** *p* < 0.0001). Abbreviations: DIVs, days in vitro; GluN2B, glutamate ionotropic receptor NMDA type subunit 2B; H, hemin; HbAA, homozygous mice expressing normal human hemoglobin A; HbSS, homozygous sickle cell mice expressing human sickle cell hemoglobin; PSD 95, postsynaptic density protein 95; TNF or T, tumor necrosis factor-alpha.

To assess the impact of genotype, developmental stage, and a simulated sickle cell microenvironment on postsynaptic organization, hippocampal neurons were isolated from male control and sickle cell neonatal mice and cultured under vehicle or TNF-α + hemin treatment conditions. Neurons were immunostained for PSD 95 and GluN2B, and colocalization was quantified at DIV 7 and DIV 14 using Pearson’s correlation coefficient, reflecting the spatial alignment of excitatory postsynaptic scaffolds and NMDA receptor subunits (Figure 4).

At DIV 7, control and sickle-cell-treated neurons exhibited a clear genotype-specific difference in PSD 95 and GluN2B colocalization (*p* < 0.01). Exposure to TNF-α and hemin significantly increased colocalization in sickle cell neurons compared to the vehicle (*p* < 0.0001) and also exceeded levels in control vehicle-treated neurons at the same stage (*p* < 0.01), suggesting that the simulated sickle cell microenvironment transiently enhances postsynaptic protein alignment, potentially via sickle cell microenvironment-induced pathways.

By DIV 14, PSD 95 and GluN2B colocalization had increased in both genotypes under vehicle conditions, reflecting expected developmental maturation. However, a clear difference persisted between control and sickle cell neurons, indicating sustained genotype-related effects. Treatment with TNF-α + hemin at DIV 14 significantly increased colocalization in sickle cell neurons relative to the vehicle (*p* < 0.05), though the effect was less pronounced than at DIV 7 and did not differ significantly from vehicle control levels.

### 3.4. PSD 95 Distribution in Hippocampal Neurons in Culture

**Figure 5:**
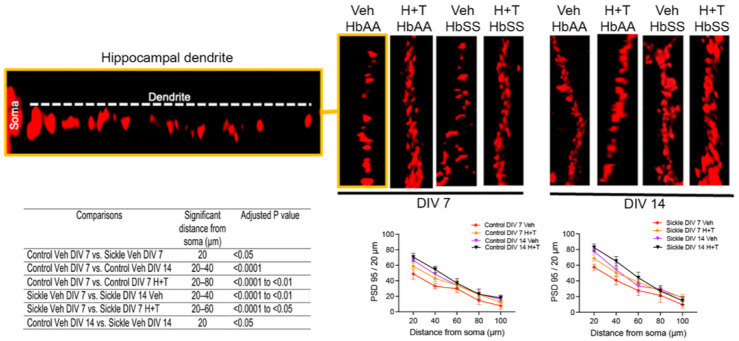
PSD 95 puncta expression in control HbAA and sickle cell HbSS mice-derived primary hippocampal neurons across DIV 7 and 14. Primary hippocampal neurons were isolated from male control and sickle cell pups (postnatal day 1). Neurons were immunostained for PSD 95 (red), a postsynaptic density marker, and imaged at ×63 oil immersion, with z-step = 0.5 µm at DIV 7 and DIV 14. Representative images show PSD 95 puncta localization in dendrites at increasing distances from the soma. PSD95 puncta were quantified and analyzed across the full 100 µm dendritic segment from the soma at 20 µm intervals. Each image represents reproducible images from three different mice (*n* = 3 mice/genotype), with 3 images acquired per mouse (9 images/condition). Values for 3 images per mouse were averaged and the mean from each was used as one value. Statistical comparisons were conducted using two-way ANOVA with Tukey’s multiple comparisons test. Data are presented as mean ± SEM. Abbreviations: DIVs, days in vitro; H, hemin; HbAA, homozygous mice expressing normal human hemoglobin A; HbSS, homozygous sickle cell mice expressing human sickle cell hemoglobin; PSD 95, postsynaptic density protein 95; T, tumor necrosis factor-alpha.

To assess genotype- and treatment-dependent effects on synaptic density, hippocampal neurons from male control and sickle cell mice were cultured under vehicle or H+T conditions and immunostained for PSD 95 at DIV 7 and DIV 14 (Figure 5). At DIV 7, control vehicle neurons exhibited a significant reduction in PSD 95 puncta compared with the sickle cell vehicle (*p* < 0.05), while H+T treatment further increased puncta density in both control and sickle cell neurons (*p* < 0.0001). Control neurons also showed a developmental increase in puncta density from DIV 7 to DIV 14 (*p* < 0.0001), and a similar increase was observed in sickle cell neurons over this interval (*p* < 0.0001). At DIV 14, sickle cell neurons retained higher PSD 95 puncta than controls (*p* < 0.05), and H+T treatment led to a gain in puncta in both groups.

## 4. Discussion

This study introduces a simplified and reproducible protocol for isolating and culturing primary hippocampal and cortical neurons from postnatal one-day-old pups of HbSS-BERK mice. We used a humanized transgenic model of SCD that closely mimics the human phenotype [32]. Existing neuronal culture methods are primarily optimized for embryonic tissue [25,33,34], and are often unsuitable for transgenic disease models that exhibit early postnatal mortality, high oxidative burden, and systemic inflammation. Our protocol addresses this critical gap by enabling long-term survival and morphological maturation of neurons from genetically fragile models, making it broadly adaptable for other conditions involving developmental, metabolic, or hemolytic stress.

What makes this approach unique is the successful adaptation of a postnatal culture method for a disease model that typically shows pronounced genetic fragility. Humanized SCD mouse strains show poor pregnancy outcomes, including high rates of fetal loss, resorption, and perinatal mortality, highlighting the fragility of these litters and the unreliability of embryonic harvesting methods [35]. Unlike genetic neurodevelopmental or neurodegenerative models such as Alzheimer’s, Parkinson’s, or Fragile X syndrome, which generally permit embryonic or early postnatal neuronal isolation because the offspring remain viable at birth, HbSS-BERK sickle cell mice experience substantial prenatal and perinatal mortality and severe systemic pathology. SCD mice also show oxidative stress, neuroinflammation, and early multiorgan involvement that extend to the central nervous system [10,36,37,38]. These vulnerabilities make standard embryonic neuronal protocols incompatible with this model and necessitate postnatal phenotypic confirmation to ensure accurate selection of homozygous sickle cell pups. Previous in vivo studies have demonstrated hippocampal neurodegeneration and cognitive and behavioral deficits in sickle cell mice [7], but few efforts have succeeded in developing a primary neuron culture system from this model that supports reliable neuronal survival, consistent synaptogenesis, and reproducible protein expression analysis over time.

A key advantage of this method is its simplified workflow relative to standard embryonic protocols. Embryonic culture methods require timed pregnancies, embryo harvesting, and neuronal isolation from tissue that cannot be phenotyped [33,34,39], making them incompatible with disease models that have high in utero mortality or require postnatal genotype confirmation [32,40]. By using phenotype-confirmed postnatal (P1) pups, our approach eliminates the need for embryonic collection and ensures reliable selection of homozygous sickle cell offspring [7,10]. Importantly, despite this simplified workflow, neurons generated using this approach attach, extend neurites, and express synaptic markers by DIV 7, showing maturation comparable to or faster than timelines reported for embryonic neuronal cultures [41,42,43]. This demonstrates that a more accessible and disease model-compatible workflow can still yield high-quality neuronal preparations. The sequential HI and LI washes following papain digestion were incorporated to reduce residual enzyme carryover in a gradual manner and improve tissue handling, which, in our hands, helped preserve the integrity of cells from fragile postnatal sickle cell brain tissue.

Our culture system provides a controlled platform to examine neuronal development without systemic influences such as anemia, hypoxia, or peripheral immune responses. Using this approach, hippocampal and cortical neurons showed progressive neurite outgrowth, increased expression of neuronal and synaptic markers (NeuN, NF200, MAP2, VGLUT1, PSD 95, and GluN2B), and remained viable through DIV 14.

Sickle-cell-derived cortical and hippocampal neurons displayed differences in attachment and neurite extension, consistent with altered early cytoskeletal organization. This is in agreement with previous studies highlighting PSD 95 as a marker of synaptic maturation [30,42,44]. Sickle-cell-derived neurons had higher PSD 95 expression at DIV 7, which persisted until DIV 14. By DIV 14, PSD 95 levels increased in both control and sickle cell neurons, though differences between genotypes remained. Under simulated sickle cell microenvironment conditions, PSD 95 expression at DIV 7 was elevated in sickle cell neurons, indicating that stress stimuli can modify postsynaptic organization. The detection of MAP2 and VGLUT1 from DIV 3 through DIV 14 further confirms neuronal viability and maturation across the culture period. MAP2 marked a dendritic structure, while VGLUT1 reflected excitatory presynaptic terminals. VGLUT1 immunoreactivity in association with MAP2-positive neuronal processes is consistent with ongoing excitatory synaptic organization during development. PSD 95 and GluN2B colocalization showed a significant increase in simulated sickle cell microenvironment at DIV 7 and 14 compared to the vehicle in male sickle-cell pup-derived hippocampal neurons. Both genotypes exhibited developmental increases in colocalization by DIV 14. Together, these findings indicate altered postsynaptic features in sickle cell neurons under baseline culture conditions, which respond to changes in the surrounding microenvironment.

Moving forward, this culture system provides a defined platform on which to interrogate the molecular pathways contributing to synaptic deficits in SCD. Future investigations to delineate specific signaling cascades implicated in cytoskeletal remodeling, oxidative stress responses, and glutamate receptor trafficking can be undertaken using these cells [45]. Incorporating functional assays, including calcium imaging and electrophysiological recordings, will be critical to establish whether observed structural alterations correlate with disruptions in neuronal activity. Moreover, this model system enables targeted screening of pharmacologic interventions, including ROS scavengers, selective anti-inflammatory agents, and compounds that stabilize synaptic architecture, to evaluate their efficacy in rescuing developmental and synaptic impairments in sickle cell neurons. Importantly, cell-to-cell interactions, such as glial neuronal neuroimmune interactions, can be facilitated using these neuronal cells. A limitation of the present study is that neuronal purity, residual glial content, preparation success rate, and culture yield were not systematically quantified. Although Ara-C was used to reduce proliferation of non-neuronal cells, residual glial populations may still have been present in culture. Therefore, this method should be interpreted as a feasibility-focused adaptation for fragile postnatal sickle-cell mice-derived brain cells rather than a fully validated purity-optimized neuronal culture protocol.

## 5. Conclusions

This study establishes a practical and versatile neuron culture method using the brains of fragile humanized SCD and control postnatal day 1 pups from mice of both sexes. Using male-pup-derived hippocampal neurons, we demonstrate that these cells respond to changes in the sickle cell microenvironment. Incubation with TNF-α and heme to create a sickle cell microenvironment led to an increase in PSD95 spine density and colocalization of GluN2B with PSD95. These in vitro findings, though preliminary, suggest an increase in glutamatergic signaling, which is observed in patients with SCD. The findings underscore intrinsic neuronal vulnerabilities in SCD and offer a tractable in vitro system to investigate how early-life inflammation and oxidative stress contribute to long-term neurocognitive and sensory dysfunction. Although neuronal purity, residual glial content, and culture yield were not systematically quantified, Ara-C treatment was used to reduce proliferation of dividing non-neuronal cells, and the present study establishes the feasibility of maintaining postnatal neuronal cultures from this fragile sickle cell model. Our protocol simplifies this workflow, and this approach produces neurons that attach, extend neurites, and express synaptic markers by DIV 7, showing maturation comparable to or faster than timelines reported for embryonic culture protocols.

## Data Availability

The data supporting the conclusions of this article is available in this manuscript. All data are available with approval from the author.

## Supplementary Materials

The following supporting information can be downloaded at https://www.mdpi.com/article/10.3390/cells15110976/s1. Table S1: Primary and secondary antibodies for neuronal markers; Table S2: ANOVA results; Figure S1: Morphological maturation of hippocampal and cortical neurons from control and sickle mice across DIV 3, 7, and 14; Figure S2: NeuN and NF200 immunoreactivity of neuronal cells at DIV 3, 7, and 14; Figure S3: MAP2 and VGLUT1 immunoreactivity of hippocampal and cortical neurons at DIV 3, 7, and 14 from male control HbAA and sickle HbSS mice.

## Abbreviations

The following abbreviations are used in this manuscript:

APV	DL-2-Amino-5-Phosphonopentanoic Acid
Ara-C	Cytosine β-D-Arabinofuranoside Hydrochloride
BSA/TI	Bovine Serum Albumin/Trypsin Inhibitor
CM+	Conditioned Medium Plus (with FBS and supplements)
CM-	Conditioned Medium Minus (without FBS)
DAPI	4′,6-Diamidino-2-Phenylindole
DIVs	Days In Vitro
DS	Dissection Solution
EDTA	Ethylenediaminetetraacetic Acid
FBS	Fetal Bovine Serum
GluN2B	Glutamate [NMDA] Receptor Subunit Epsilon-2
HBSS	Hank’s Balanced Salt Solution
HbAA	Homozygous Humanized Mice Expressing Hemoglobin A
HbSS	Homozygous Humanized Sickle Cell Mice Expressing Hemoglobin S
HEPES	N-(2-Hydroxyethyl) Piperazine-N′-(2-Ethanesulfonic Acid)
HI	High Inhibitor Solution
LI	Low Inhibitor Solution
MAP2	Microtubule-Associated Protein 2
NBM/B27	Neurobasal Medium with B27 Supplement
NeuN	Neuronal Nuclei Marker (RBFOX3)
NF200	Neurofilament 200 kDa
NMDAR	N-Methyl-D-Aspartate Receptor
P1	Postnatal Day 1
PBS	Phosphate-Buffered Saline
PSD 95	Postsynaptic Density Protein 95
RT	Room Temperature
SCD	Sickle Cell Disease
TNF-α	Tumor Necrosis Factor-alpha
VGLUT1	Vesicular Glutamate Transporter 1
