# Supplementary material for: Simplified Procedure for Isolation and Culture of Neuronal Cells from Brains of Sickle Cell Mice
Source: Cells. 2026 May 26;15(11):976. doi: 10.3390/cells15110976 (PMC13256901; doi:10.3390/cells15110976)
Supplement: Supplementary file 1 [file cells-15-00976-s001.zip › cells-4186050-supplementary.pdf]

Supplemental file

Antigen	Primary antibody (host, supplier, catalog no.)	Dilution / incubation	Secondary antibody (host, catalog no.)	Dilution / incubation
RBFOX3 (NeuN)	Goat anti-NeuN (Novus Biologicals, NBP3-05554)	1:200, overnight at 4°C	Cy2 donkey anti-goat (Jackson ImmunoResearch, 705-225-147)	1:200, 1 h at room temperature
NF200	Chicken anti-NF200 (Neuromics, CH22104)	1:3000, overnight at 4°C	Cy5 donkey anti-chicken (Jackson ImmunoResearch, 703-175-155)	1:200, 1 h at room temperature
PSD-95	Guinea pig anti-PSD-95 (Alomone Labs, APZ-009-GP)	1:500, overnight at 4°C	Cy5 donkey anti-guinea pig (Jackson ImmunoResearch, 706-175-148)	1:200, 1 h at room temperature
GluN2B	Rabbit anti-GluN2B (Alomone Labs, AGC-003)	1:500, overnight at 4°C	Cy2 donkey anti-rabbit (Jackson ImmunoResearch, 711-225-152)	1:200, 1 h at room temperature
MAP2	Chicken anti-MAP2 (Thermo Fisher Scientific, PA1-1005)	1:2000, overnight at 4°C	Cy5 donkey anti-chicken (Jackson ImmunoResearch, 703-175-155)	1:200, 1 h at room temperature
VGLU1	Rabbit anti-VGLUT1 (Cell Signaling Technology, 47181)	1:300, overnight at 4°C	Cy2 donkey anti-rabbit (Jackson ImmunoResearch, 711-225-152)	1:200, 1 h at room temperature

Supplemental Table S1. Primary and Secondary Antibodies for Neuronal Markers. The table lists the target antigen, primary antibody source and catalog number, antibody dilution, and incubation conditions, along with the corresponding secondary antibodies and their dilution and incubation conditions.

**Fig. 4. Colocalization of PSD95 and GluN2B in Male HbSS Mice Primary Hippocampal Neurons Two-way ANOVA**					
			Alpha	*0.05*	
Source of Variation	*% of total variation*	*P value*	*P value summary*	*Significant?*	
Interaction	1.214	0.9875	ns	No	
Row Factor	0.02985	0.9694	ns	No	
Column Factor	81.48	< 0.0001	****	Yes	
ANOVA table	*Sum Squared (SS)*	*Degrees of Freedom (DF)*	*Mean Squared (MS)*	*F (DFn, DFd)*	*P value*
Interaction	0.01129	10	0.001129	"F (10, 36) = 0.2529"	P=0.9875
Row Factor	0.0002778	2	0.0001389	"F (2, 36) = 0.03110"	P=0.9694
Column Factor	0.7583	5	0.1517	"F (5, 36) = 33.96"	P < 0.0001
Residual	0.1608	36	0.004465		
Total	0.9307	53			
**Fig. 5. Total PSD95 Puncta Counts at 100 µm From the Soma in Male HbSS Mice Primary Hippocampal Neurons Two-way ANOVA**					
			Alpha	*0.05*	
Source of Variation	*% of total variation*	*P value*	*P value summary*	*Significant?*	
Interaction	3.475	< 0.0001	****	Yes	
Time	79.74	< 0.0001	****	Yes	
Treatment	10.45	< 0.0001	****	Yes	
ANOVA table	*Sum Squared (SS)*	*Degrees of Freedom (DF)*	*Mean Squared (MS)*	*F (DFn, DFd)*	*P value*
Interaction	2718	20	135.9	F (20, 150) = 4.112	P < 0.0001
Time	62374	4	15593	F (4, 150) = 471.8	P < 0.0001
Treatment	8172	5	1634	F (5, 150) = 49.45	P < 0.0001
Residual	4958	150	33.05		
Total	78222	179			

Supplemental Table S2. ANOVA Results. Statistical analyses showing two-way ANOVA results for Figure 4, PSD95 and GluN2B colocalization and Figure 5, total PSD95 puncta counts in male HbSS primary hippocampal neurons. Data include source of variation, percent total variation, F values, degrees of freedom, mean squared values, and significance levels.

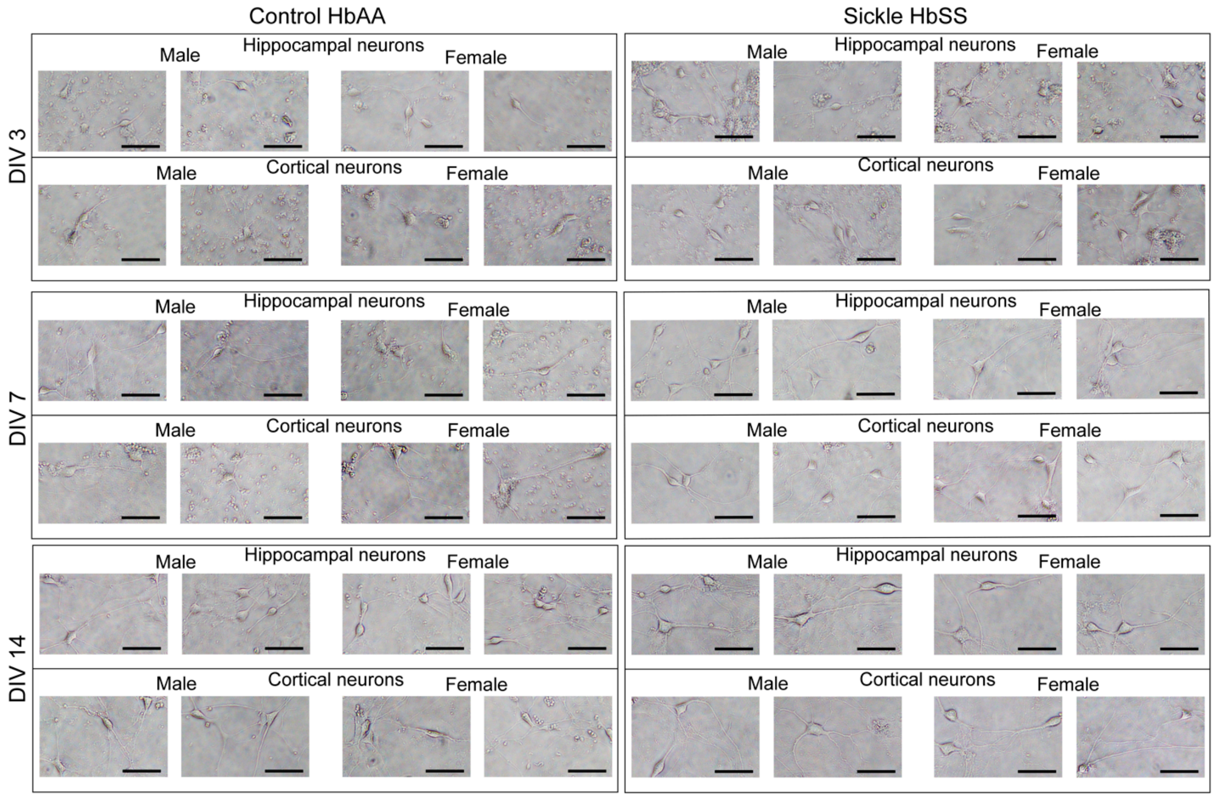

Supplemental Figure S1. Morphological maturation of hippocampal and cortical neurons from control and sickle mice across DIV 3, 7, and 14. Representative brightfield images of live primary hippocampal and cortical neurons isolated from control and sickle neonatal mice (postnatal day 1) are shown. Compared with DIV3, neurons at DIV14 qualitatively showed a more extensive cellular network, with increased axonal growth and branching across both genotypes and brain regions. Magnification ×20, Scale bar: 50 µm. Each image represents cells from 3 different mice per genotype. Abbreviations: DIV, days in vitro; HbAA, homozygous mice expressing normal human hemoglobin A; HbSS, homozygous sickle mice expressing human sickle hemoglobin.

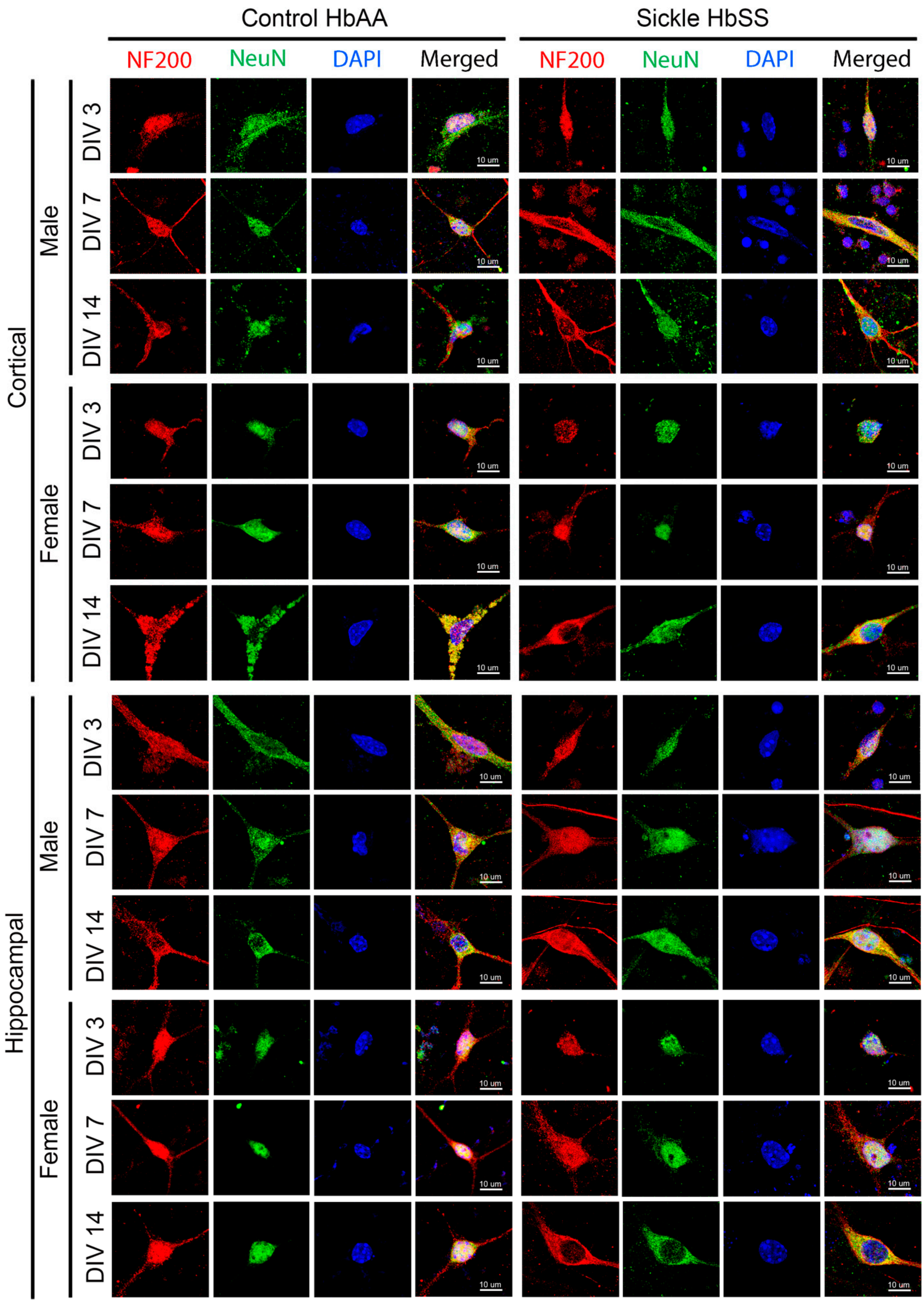
Control HbAA
Sickle HbSS
NF200
NeuN
DAPI
Merged
NF200
NeuN
DAPI
Merged
Cortical
Male
Female
Hippocampal
Male
Female
DIV 3
DIV 7
DIV 14
10 um

Supplemental Figure S2. NeuN and NF200 immunoreactivity of neuronal cells at DIV 3, 7, and 14. Primary hippocampal and cortical neurons were isolated from male and female control HbAA and sickle HbSS neonatal mice (postnatal day 1) and immunostained for NeuN (green), a neuronal marker, and NF200 (red), a neurofilament protein marking axons, and counterstained with DAPI (blue) to visualize nuclei. Each image represents reproducible images from three different mice, with 3 images acquired per mouse (9 images/condition). Confocal images were acquired with ×63 objective with ×2.3 digital zoom, z-stacks (0.5 μm step size). Scale bar: 20 μm. Abbreviations: DIV, days in vitro; HbAA, homozygous mice expressing normal human hemoglobin A; HbSS, homozygous sickle mice expressing human sickle hemoglobin; NeuN, neuronal nuclei (RBFOX3); NF200, neurofilament 200 kDa.

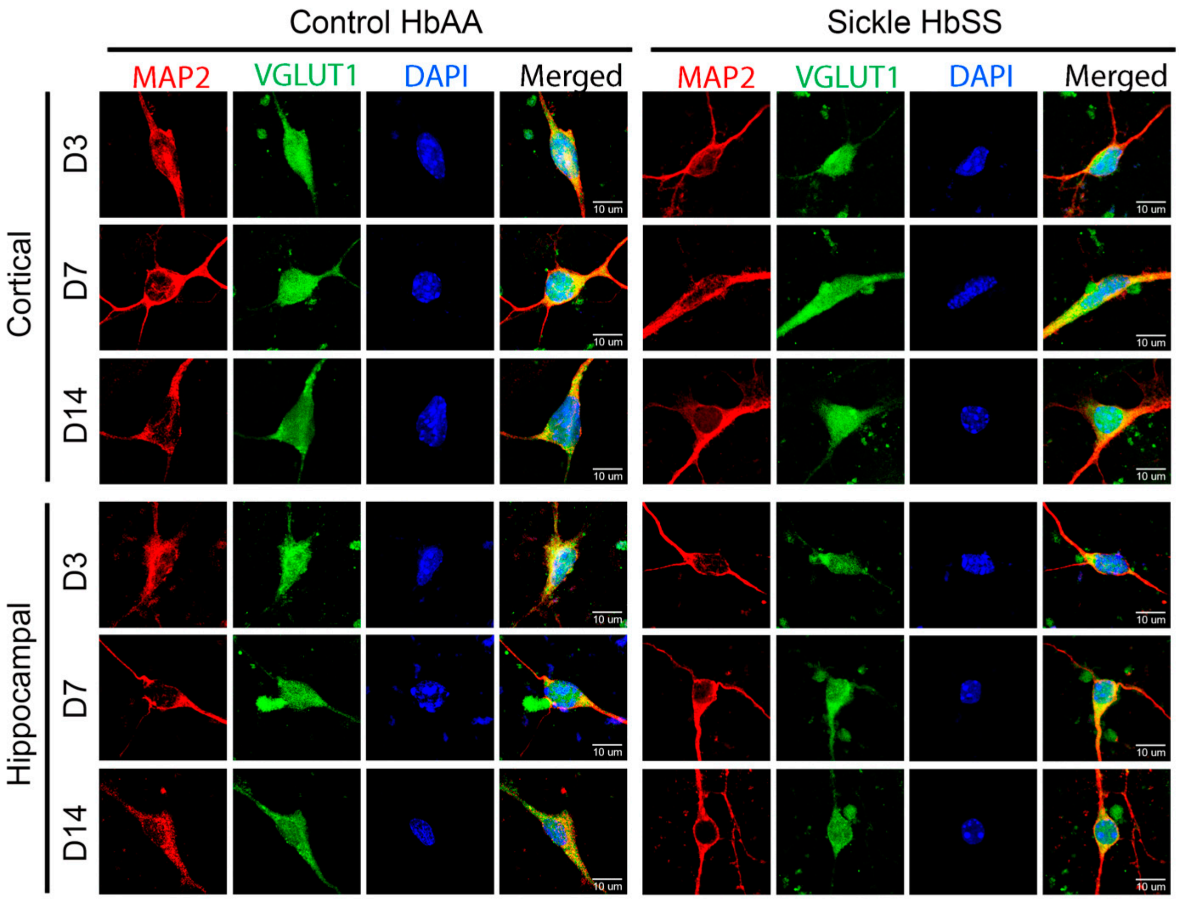

Supplemental Figure S3. MAP2 and VGLUT1 immunoreactivity of hippocampal and cortical neurons at DIV 3, 7, and 14 from male control HbAA and sickle HbSS mice. Primary hippocampal and cortical neurons were isolated from male control and sickle neonatal mice (postnatal day 1). Neurons were immunostained for VGLUT1 (green), a presynaptic marker of glutamatergic vesicles, and MAP2 (red), a dendritic marker. Confocal imaging was performed using a ×63 oil immersion objective with 0.5 µm z-step. Each image represents reproducible images from three different mice (n = 3 mice/genotype), with 3 images acquired per mouse (9 images/condition). Abbreviations: DIV, days in vitro; HbAA, homozygous mice expressing normal human hemoglobin A; HbSS, homozygous sickle mice expressing human sickle hemoglobin; MAP2, microtubule-associated protein 2; VGLUT1, vesicular glutamate transporter 1.
